# The hospitalization burden of all-cause pneumonia in China: A population-based study, 2009–2017

**DOI:** 10.1016/j.lanwpc.2022.100443

**Published:** 2022-04-06

**Authors:** Yizhen Hu, Yuting Han, Canqing Yu, Yu Guo, Pei Pei, Ling Yang, Yiping Chen, Huaidong Du, Dianjianyi Sun, Yuanjie Pang, Wenbin Niu, Sushila Burgess, Alex Hacker, Junshi Chen, Zhengming Chen, Jun Lv, Liming Li, China Kadoorie Biobank Collaborative Group

**Affiliations:** a Department of Epidemiology and Biostatistics, School of Public Health, Peking University, Xueyuan Road, Haidian District, Beijing 100191, China; b Peking University Center for Public Health and Epidemic Preparedness and Response, Beijing 100191, China; c Fuwai Hospital Chinese Academy of Medical Sciences, Beijing 100037, China; d Chinese Academy of Medical Sciences, Beijing 100730, China; e Medical Research Council Population Health Research Unit at the University of Oxford, Oxford OX3 7LF, UK; f Clinical Trial Service Unit and Epidemiological Studies Unit, Nuffield Department of Population Health, University of Oxford, Oxford OX3 7LF, UK; g Maiji District Center for Disease Control and Prevention, Gansu 741020, China; h China National Center for Food Safety Risk Assessment, Beijing 100022, China; i Key Laboratory of Molecular Cardiovascular Sciences (Peking University), Ministry of Education, Beijing 100191, China

**Keywords:** Pneumonia, Epidemiology, Hospitalization, Case fatality rate, Length of stay, Adult

## Abstract

**Background:**

Pneumonia represents a public health problem of substantial health and economic burden. However, the evidence on the burden of adult pneumonia is limited in China.

**Methods:**

The China Kadoorie Biobank recruited 512,725 participants aged 30–79 years from five urban and five rural areas during 2004–2008. The current analyses included 506,086 participants who were alive in 2009. Pneumonia hospitalizations were ascertained through the health insurance system until December 31, 2017. Generalized linear models were used to examine the secular trends and regional and population variations in pneumonia hospitalization rate, mean length of hospital stay (LOS), and 30-day case fatality rate (CFR).

**Findings:**

A total of 27,879 participants with 36,567 pneumonia hospitalizations were identified with a mean follow-up time of 8·9 years. The unadjusted hospitalization rate was 8·4 (95% confidence interval [CI]: 8·3, 8·6) per 1000 person-years, with an increase of 15·5% annually from 4·2 (3·9, 4·4) in 2009 to 10·9 (10·6, 11·3) in 2017, after adjusting for age, sex, study area. The mean LOS was 8·8 (95% CI: 8·7, 8·9) days, with a slight decrease of 1·0% annually from 2009 to 2017. The average 30-day CFR remained practically unchanged at 2·4 (95% CI: 2·2, 2·5) deaths per 100 admissions. A clear seasonal pattern of pneumonia hospitalization rate was observed, and the hospitalization rate and CFR differed across regions and subpopulations of different ages and underlying conditions.

**Interpretation:**

There was an increasing hospitalization burden of pneumonia in Chinese adults, especially for adults aged ≥60 years or those with underlying conditions.

**Funding:**

The National Natural Science Foundation of China, the Kadoorie Charitable Foundation, the National Key R&D Program of China, the Chinese Ministry of Science and Technology.

**Translated abstract in Chinese:**

This translation in Chinese was submitted by the authors and we reproduce it as supplied. It has not been peer reviewed. Our editorial processes have only been applied to the original abstract in English, which should serve as reference for this manuscript.

**摘要**

**背景:**肺炎是一个重要的公共卫生问题, 威胁人群健康的同时还造成沉重的经济负担.然而, 基于人群研究的中国成年人肺炎住院负担的研究证据十分有限.

**方法:**中国慢性病前瞻性研究(China Kadoorie Biobank)于2004-2008年募集了来自5个城市和5个农村地区的50余万30-79岁的成年人.本研究纳入506,086名在2009年1月1日仍存活的研究对象.研究通过链接医保数据库获取研究对象2009年1月1日至2017年12月31日的肺炎住院数据.采用广义线性模型分析肺炎住院率,肺炎住院患者的住院时长及30天病死率的长期趋势及其地区,人群分布情况.

**结果:**在平均8.9年的随访期内, 27,879名研究对象发生肺炎住院, 合计发生肺炎住院36,567人次.研究期间内肺炎的粗住院率为8.4 (95% CI:8.3, 8.6)/1000人年, 在调整年龄,性别和地区后, 由2009年的4.2 (3.9, 4.4)增长至2017年的10.9 (10.6, 11.3), 平均年增长率为15.5%.研究期间肺炎住院患者平均住院时长为8.8(95% CI:8.7, 8.9)天, 平均年缩短率为1.0%.肺炎住院患者的30天病死率没有明显改变, 研究期间粗病死率为2.4(95% CI:2.2, 2.5)/100例肺炎住院.本研究还发现肺炎住院率存在明显的季节性;不同地区,年龄和基础疾病的患者, 肺炎住院率和30天病死率都存在差异.

**解读:**中国成年人中肺炎住院负担重且呈现增长趋势, 尤其是在≥60岁或存在基础疾病的研究对象中.

**Research in context**

**Evidence before this study**

We searched PubMed for articles that documented the burden and epidemiological features of adult pneumonia published before February 18, 2022, using the search terms “pneumonia” OR “epidemiology” OR “burden” OR “adult”. We manually searched reference lists and retrieved articles as well. Previous studies were mostly conducted in Western populations and have shown mixed secular trends in pneumonia hospitalization rates and case-fatality rates since 1980s among adults. Ongoing reliable estimates of pneumonia incidence are essential for evaluating the demand for preventive measures and healthcare resources. However, the data on the trends in the burden of adult pneumonia from population-based studies, covering a broader spectrum of populations, are lacking in China.

**Added value of this study**

In the present large populational-based prospective cohort study of Chinese adults, we found a gradually increasing trend in pneumonia hospitalization rate from 2009 to 2017, even after adjusting for the ageing of participants and the growing burden of underlying conditions. The 30-day case-fatality rate remained unchanged. The hospitalization rate and case-fatality rate differed across regions and subpopulations of different ages and underlying conditions. Our study provided parameters to help determine needs for preventive measures and medical resources and parameters for health economics assessments.

**Implications of all the available evidence**

The recommendation of proven prevention measures for adult pneumonia should be reinforced, particularly in high-risk groups, considering the comparatively higher healthcare cost and the poorer outcome.

Alt-text: Unlabelled box

## Introduction

Pneumonia remains a leading infectious cause of hospitalization and death among adults,^1^^,^^2^ with a high mortality rate in patients requiring hospitalization.^3^^,^^4^ The hospitalization rate of pneumonia increases sharply with advancing age,^5^, ^6^, ^7^, ^8^, ^9^ and the number of underlying conditions.^6^^,^^9^ As shown in previous studies conducted in Western populations,^6^^,^^10^, ^11^, ^12^, ^13^ pneumonia hospitalization increased with the rapidly ageing population and the dramatic increase in chronic disease prevalence. If so, the increasing burden of pneumonia hospitalization could impose a substantial clinical and economic burden and also contribute to the deteriorating crisis of antibiotic resistance.^3^

Ongoing reliable estimates of pneumonia incidence are essential for evaluating the demand for preventive measures (e.g., vaccination) and healthcare resources. Current hospital-based surveillance and research for adult community-acquired pneumonia (CAP) in China were mostly focused on clinical features and aetiology.^14^, ^15^, ^16^ The reasonably accurate estimates of the hospitalization rate are usually unavailable due to the lack of well-defined catchment populations. In China, data on the burden of adult pneumonia from population-based studies are lacking besides two studies conducted in urban areas,^5^^,^^17^ restricted to a specific calendar year or a single city.^5^^,^^17^ Understanding the epidemiological features of pneumonia could help assess the potential health benefits of preventive measures targeting individuals of different characteristics and identify public health priorities. Besides, the length of hospital stay (LOS) and case-fatality rate (CFR) for pneumonia are important parameters in relevant health economic studies. However, there are still evidence gaps needed to address for Chinese populations.

This study aimed to examine the secular trend and regional and population variations in pneumonia hospitalization from 2009 to 2017, using data from a nationwide cohort of Chinese adults. In particular, we hoped to identify subpopulations that are most likely to benefit from prevention measures such as vaccination and provide basic parameters for relevant health economic evaluation.

## Methods

### Study design and participants

We used data from the China Kadoorie Biobank, a prospective cohort study that enrolled 512,725 participants aged 30–79 years from 5 urban (Harbin, Qingdao, Suzhou, Liuzhou, and Haikou) and 5 rural (Gansu, Henan, Zhejiang, Hunan, and Sichuan) areas across China during 2004–2008. A detailed description of the design, methods, and procedures has been previously published.^18^^,^^19^ Briefly, at baseline, all participants provided written informed consent and completed an interviewer-administered electronic questionnaire, also had physical examinations and blood samples collected for long-term storage. Long-term follow-up data on cause-specific mortality and major morbidities, as well as episodes of hospital admission, were obtained by electronic linkage via unique personal identification numbers to the national Disease Surveillance Points (DSP) system,^20^ local disease registry, and health insurance (HI) claim system, or by active follow-up. All disease diagnoses and underlying causes of death were coded according to the 10th revision of the International Classification of Diseases (ICD-10) by trained staff blinded to the baseline information. By the end of December 31, 2017, only 5301 (1.0%) participants were lost to follow-up. The Ethical Review Committee of the Chinese Center for Disease Control and Prevention (Beijing, China) and the Oxford Tropical Research Ethics Committee, University of Oxford (UK) approved the study.

### Ascertainment of pneumonia hospitalization

Hospital admissions for pneumonia were identified using ICD-10 codes J12-J18. The study outcomes of the present analysis were the pneumonia hospitalization rate (per 1000 person-years), LOS (in days), and 30-day CFR (per 100 admissions) for pneumonia. The first two indicators mainly reflected the healthcare use of pneumonia hospitalization, and the 30-day CFR was commonly used to measure the short-term outcome of pneumonia.^11^^,^^12^ The LOS was defined as the consecutive days between the admission date and the discharge date of the hospitalization. Day-case admissions (i.e., patients admitted and discharged on the same day) were defined as having a LOS of 0·5 days. The 30-day CFR was defined as all-cause death within 30 days from the admission date for the first-ever event for pneumonia.

### Assessment of covariates

Dedicated staff used a laptop-based questionnaire at baseline to collect data on sociodemographic characteristics (e.g., age, sex, education attainment), lifestyle factors (e.g., physical activity, tobacco smoking, alcohol consumption), and personal medical history, including hypertension, diabetes, ischemic heart disease, stroke, chronic obstructive pulmonary disease (COPD), tuberculosis, asthma, chronic kidney disease, cirrhosis/chronic hepatitis, and cancer. Physical measurements were measured using calibrated instruments following standard procedures, including height and weight (further derived as body mass index), blood pressure, and lung function. Random plasma glucose was measured on-site immediately. Prevalent hypertension, diabetes, and COPD at baseline were defined based on the self-reported clinician diagnosis and baseline physical measurements.^21^^,^^22^ During long-term follow-up, the disease status of the abovementioned ten underlying conditions was updated through established registries and the HI claim system.

The HI schemes of participants were identified annually during 2012–2016. In line with the previous study,^23^ the HI scheme for years with missing data was imputed based on the closest scheme available. Since both the urban resident basic medical insurance (URBMI) and the new rural cooperative medical scheme (NRCMS) provided similar benefits, and the two schemes in four of the ten study areas were merged into a single scheme from 2012 to 2013, we, therefore, combined these two schemes. The HI scheme was categorized into three groups: urban employee basic medical insurance (UEBMI), URBMI or NRCMS, and uninsured. The target population, administration, source and level of funding for the scheme, and benefits varied substantially between UEBMI and URBMI/NRCMS.^24^

### Statistical analysis

The recruitment of 512,725 participants to the CKB was completed on July 15, 2008. The linkage to the HI system for all study areas was completed until 2009. We, therefore, restricted the study period to 2009 onward. In the analysis of pneumonia hospitalization rate, participants who died (*n* = 6424) or were lost to follow-up before 2009 (*n =* 215) were excluded, leaving 506,086 participants contributing to the analysis. In the analysis of LOS, hospitalization records with missing or implausible discharged dates (*n =* 643) or with implausible LOS (greater than 99th percentile, that is 35 days, or less than 0, *n =* 348) were excluded, leaving 27,292 participants with 35,576 records for analysis. In the analysis of 30-day CFR, participants with a history of pneumonia before 2009 were excluded (*n =* 419), leaving 27,460 participants for analysis.

Comparisons of background characteristics between participants with or without pneumonia hospitalization during follow-up were made using the Chi-Squared test for categorical variables and one-way analysis of variance (ANOVA) F-test for continuous variables. The pneumonia hospitalization rate was estimated using generalized linear models (GLM) with negative binomial distribution and a log link function.^23^ The annual mean LOS per admission was estimated using GLM with gamma distribution and a log link function. The annual 30-day CFR was estimated using GLM with binomial distribution and a logit link function. We used cluster-adjusted robust standard errors to account for the repeat hospital admissions for pneumonia within individuals during follow-up in the analyses of pneumonia hospitalization and LOS.

The secular trends in pneumonia hospitalization rate, mean LOS, and 30-day CFR over the study period were examined using the corresponding GLM mentioned above, with adjustment for sex, study area, and annually updated age where appropriate. We also performed subgroup analyses according to urban or rural areas, geographic locations, age groups, sex, HI schemes, and type and number of underlying conditions. The age and disease status of participants were updated at the start of each year from 2009 to 2017 in the analysis of hospitalization rate or until the occurrence of the index hospitalization in the analysis of LOS and 30-day CFR.

Sensitivity analyses were performed to check the robustness of the findings. When examining the overall secular trend, regional, and population variations in hospitalization rate, mean LOS, and 30-day CFR, we further adjusted for individual socioeconomic, lifestyle, and annually updated underlying condition variables to exclude their potential contributions. Direct standardization was also applied in the secular trend analysis, using age, sex, and regional distribution of the overall CKB population in 2009. To exclude the potential bias caused by hospital readmissions or hospital-acquired pneumonia, we restricted analysis in the first-ever admission for pneumonia during follow-up or participants without any other hospital admission in the previous 30 days.^13^ All analyses were performed by using Stata, version 15.0. A two-tailed *P*-value < 0.05 was considered significant.

### Role of the funding source

The funders had no role in the study design, data collection, data analysis and interpretation, writing of the report, or the decision to submit the article for publication.

## Results

### Participant characteristics

During a mean follow-up of 8·6 (SD 1·4) years from 2009 to 2017 (with 4,362,844 person-years), a total of 27,879 participants with 36,567 pneumonia hospitalizations were identified, of whom 5653 (20·3%) participants suffered from recurrent (≥2) pneumonia hospitalizations. The mean age (SD) of included participants at the beginning of 2009 was 53·7 (10·6), 40·8% were male, and 55·7% were rural residents (Table 1). Participants hospitalized for pneumonia were more likely to be older and live in rural areas. Compared to participants without pneumonia hospitalization, those hospitalized for pneumonia had a higher prevalence of underlying conditions except hypertension. Age, male, residents of South or rural areas, and number of underlying conditions were further increased with increased pneumonia hospitalizations during the study period (Supplementary Table 1).

**Table 1:** Background characteristics of participants by pneumonia hospitalization during 2009–2017.

	Overall (*N* = 506 086)	Pneumonia hospitalization^a^		*P*-value
		Absence (*n* = 478 207)	Presence (*n* = 27 879)	
**Age at the beginning of 2009, year (SD)**	53.7 (10.6)	53.4 (10.5)	59.7 (10.5)	<0.001
**Age groups at the beginning of 2009, *n* (%)**				<0.001
<60 years	356206 (70.4)	342852 (71.7)	13354 (48.3)	
60–69 years	100065 (19.8)	91819 (19.2)	8246 (29.2)	
≥70 years	49815 (9.8)	43536 (9.1)	6279 (22.5)	
**Sex, *n* (%)**				0.161
Male	206238 (40.8)	194261 (40.7)	11977 (41.4)	
Female	299848 (59.2)	283946 (59.3)	15902 (58.6)	
**Region, *n* (%)**				<0.001
Urban	224132 (44.3)	212873 (44.6)	11259 (38.3)	
Rural	281954 (55.7)	265334 (55.4)	16620 (61.7)	
**Geographic location**^b^**, *n* (%)**				<0.001
Northern China	203280 (40.2)	195540 (40.8)	7740 (29.1)	
Southern China	302806 (59.8)	282667 (59.2)	20139 (70.9)	
**Education, *n* (%)**				<0.001
No formal school	92991 (18.4)	86913 (18.3)	6078 (19.0)	
Primary or middle school	306322 (60.5)	288762 (60.5)	17560 (60.8)	
High school or above	106773 (21.1)	102532 (21.1)	4241 (20.2)	
**Health insurance scheme**^c^**, *n* (%)**				<0.001
UEBMI	186247 (37.7)	176454 (37.5)	9793 (39.7)	
URBMI or NRCMS	280881 (56.8)	263985 (56.8)	16896 (57.5)	
Uninsured	27383 (5.5)	26601 (5.7)	782 (2.7)	
**Physical activity, MET-h/day (SD)**	21.2 (13.9)	21.2 (13.9)	20.9 (12.5)	0.011
**Tobacco smoking**^d^**, *n* (%)**				<0.001
Non-current daily smoker	358403 (70.8)	339920 (70.9)	18483 (68.9)	
1–14 cig/d	54533 (10.8)	51116 (10.8)	3417 (10.8)	
15–24 cig/d	66931 (13.2)	62875 (13.2)	4056 (14.4)	
≥25 cig/d	26219 (5.2)	24296 (5.1)	1923 (6.0)	
**Alcohol drinking, *n* (%)**				0.179
Non-current daily drinker	460637 (91.0)	435737 (91.0)	24900 (91.4)	
Daily male<30/female<15 g pure alcohol	10525 (2.1)	9820 (2.1)	705 (1.9)	
Daily male≥30/female≥15 g pure alcohol	34924 (6.9)	32650 (6.9)	2274 (6.7)	
**Body-mass index**^e^**, *n* (%)**				<0.001
Underweight, <18.5 kg/m^2^	21514 (4.3)	19501 (4.1)	2013 (5.8)	
Normal, 18.5–23.9 kg/m^2^	262338 (51.8)	247985 (51.9)	14353 (51.6)	
Overweight or obesity, ≥24.0 kg/m^2^	222232 (43.9)	210719 (44.0)	11513 (42.6)	
**Waist circumference, *n* (%)**				0.956
Male<85 cm, female<80 cm	293192 (57.9)	277155 (57.9)	16037 (58.4)	
Male≥85 cm, female≥80 cm	212894 (42.1)	201052 (42.1)	11842 (41.6)	
**Prevalence of underlying conditions at the beginning of 2009, *n* (%)**				
Hypertension	179723 (35.5)	168000 (35.6)	11723 (34.7)	0.009
Diabetes	31609 (6.2)	29196 (6.2)	2413 (6.9)	<0.001
Ischemic heart disease	20860 (4.1)	18728 (4.0)	2132 (5.3)	<0.001
Stroke	13507 (2.7)	12264 (2.6)	1243 (3.0)	<0.001
COPD	36807 (7.3)	32062 (6.9)	4745 (11.2)	<0.001
Tuberculosis	7840 (1.5)	6957 (1.5)	883 (2.4)	<0.001
Asthma	3149 (0.6)	2776 (0.6)	373 (1.2)	<0.001
Chronic kidney disease	7726 (1.5)	7045 (1.5)	681 (1.9)	<0.001
Cirrhosis/chronic hepatitis	7496 (1.5)	7042 (1.5)	454 (1.6)	0.015
Cancer	5057 (1.0)	4604 (1.0)	453 (1.3)	<0.001
**Number of the above conditions at the beginning of 2009, *n* (%)**				<0.001
0	270729 (53.5)	259726 (53.7)	11003 (49.5)	
1	172746 (34.1)	162077 (34.1)	10669 (34.9)	
2	49506 (9.8)	44896 (9.6)	4610 (12.0)	
≥3	13105 (2.6)	11508 (2.5)	1597 (3.6)	

SD: standard deviation; UEBMI: urban employee basic medical insurance; URBMI: urban resident basic medical insurance; NRCMS: new rural cooperative medical scheme; MET: metabolic equivalent task; COPD: chronic obstructive pulmonary disease.

All variables were measured at baseline unless indicated otherwise.

a Data were adjusted for age at corresponding year, sex, and study area where appropriate.

b We classified the study areas in Harbin, Qingdao, Gansu, and Henan as northern China and the other areas including Haikou, Suzhou, Liuzhou, Sichuan, Hunan, and Zhejiang as southern China according to the Qinling Mountain-Huaihe River Line.

c Information on health insurance scheme is for the year 2012 (*n* = 494,511).

d Participants who quit smoking because of illness were classified as current daily smokers.

e Two participants had missing data on the body-mass index.

### Secular trend and seasonal variation in pneumonia hospitalization

**Figure 1:**
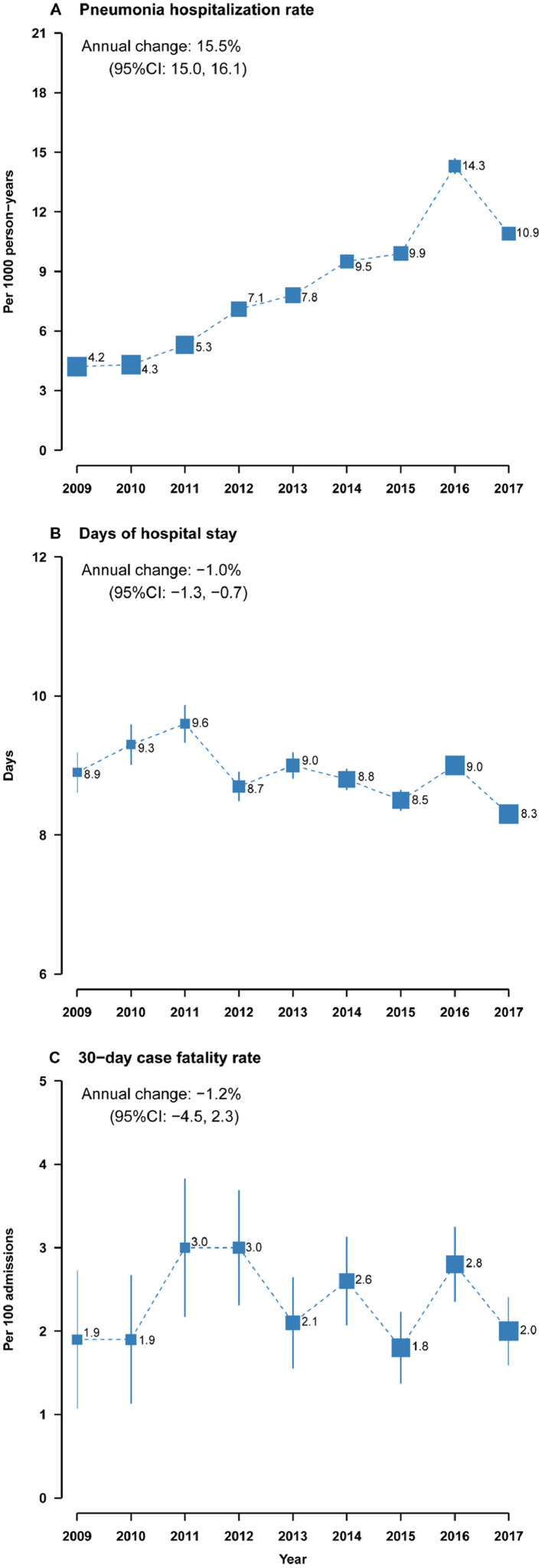
**Secular trends in pneumonia hospitalization from 2009 to 2017**. The data were adjusted for annually updated age, sex, and study area. Age was updated at the start of each year from 2009 to 2017 in the analysis of hospitalization rate or until the occurrence of the index hospitalization in the analysis of length of hospital stay and 30-day case fatality rate. The area of each square is inversely proportional to the variance, and 95% confidence intervals are shown. Numbers alongside the squares are hospitalization rates per 1000 person-years, mean length of hospital stay in days, or case fatality rates per 100 admissions, as appropriate.

The unadjusted hospitalization rate for pneumonia was 8·4 (95% confidence interval [CI]: 8·3, 8·6) admissions per 1000 person-years between 2009 and 2017, which increased by 22·2% (21·6%, 22·8%) per year from 3·3 (3·1, 3·5) in 2009 to 13·2 (12·8, 13·6) in 2017 (Supplementary Figure 1A). The annual increases in age-, sex-, and study area-adjusted hospitalization rate was 15·5% (95% CI: 15·0%, 16·1%), from 4·2 (3·9, 4·4) in 2009 to 10·9 (10·6, 11·3) in 2017 (Figure 1A). Similar results were obtained when the standardized method was applied (Supplementary Figure 1A). After full adjustment for individual socioeconomic, lifestyle, and annually updated underlying conditions, the annual increase in hospitalization rate was slightly reduced to 14·3% (95% CI: 13·8%, 14·9%).

The unadjusted mean LOS for pneumonia was 8·8 (95% CI: 8·7, 8·9) days, with a slight decrease from 8·7 (8·4, 9·0) days in 2009 to 8·6 (8·5, 8·7) days in 2017 (Supplementary Figure 1B). The age-, sex-, and study area-adjusted annualized change in LOS was -1·0% (95% CI: -1·3%, -0·7%) (Figure 1B). The unadjusted mean 30-day CFR was 2·4 (95% CI: 2·2, 2·5) deaths per 100 admissions, with an increase from 1.5 (0·9, 2·1) in 2009 to 2·4 (1·9, 2·8) in 2017 (Supplementary Figure 1C). However, after basic or full adjustments or standardization, there was no secular change in the CFR during the study period, with a basic adjusted annual change by -1·2% (95% CI: -4·5%, 2·3%) (Figure 1C and Supplementary Figure 1C).

The secular trends in hospitalization rate, LOS, and 30-day CFR for pneumonia were generally consistent across different regions and characteristics of participants, with a few exceptions (Supplementary Figures 2 and 3). The apparent decrease in the mean LOS occurred mainly in participants living in urban areas or those enrolled in the UEBMI scheme, unlike their counterparts. We also restricted the analyses in the first-ever admission for pneumonia or in participants without hospital admission in the previous 30 days (Supplementary Figures 4 and 5). Only the annual change in hospitalization rate showed a moderate reduction (12·0%; 95% CI: 11·4%, 12·5%) when restricted analysis in the first-ever admission for pneumonia.

The pneumonia hospitalization showed a clear seasonal trend from 2009 to 2017, and the variation pattern differed between the northern and southern parts of China (Supplementary Figure 6). In northern China, the hospitalization rate peaked in the colder months (December through March). In contrast, two peaks of hospitalization rate existed in southern China, with a larger peak occurring in the colder months (December through March) and a smaller peak in the warmer months (July and August).

### Regional variation in pneumonia hospitalization

Rural residents had a higher hospitalization rate (9·5 vs. 7·3 admissions per 1000 person-years) but a shorter mean LOS (7·6 vs. 10·6 days) and a lower 30-day CFR (2·0 vs. 2·8 deaths per 100 admissions) than urban residents (all *P* < 0·001) (Table 2). There were also apparent differences between Southern and Northern China, with a higher hospitalization rate (10·1 vs. 5·9), shorter LOS (8·0 vs. 10·9), and lower 30-day CFR (2·1 vs. 3·2) in Southern than Northern China (all *P <* 0·001). The adjustment for potential confounders, restriction of analysis in the first-ever hospital admission, or exclusion of participants who had any other hospital admission in the previous 30 days did not change the findings significantly (Supplementary Tables 2–4).

**Table 2:** Regional and population variations in pneumonia hospitalization.

	Pneumonia hospitalization rates per 1000 person-years		Days of hospital stay		Case-fatality rates per 100 admissions	
	Adjusted rates (95% CIs)	*P*-value	Adjusted days (95% CIs)	*P*-value	Adjusted rates (95% CIs)	*P*-value
**Region**						
Urban	7.3 (7.2, 7.5)	Ref.	10.6 (10.5, 10.7)	Ref.	2.8 (2.6, 3.1)	Ref.
Rural	9.5 (9.4, 9.7)	<0.001	7.6 (7.5, 7.7)	<0.001	2.0 (1.7, 2.2)	<0.001
**Geographic location**^a^						
Northern China	5.9 (5.8, 6.1)	Ref.	10.9 (10.8, 11.0)	Ref.	3.2 (2.8, 3.6)	Ref.
Southern China	10.1 (9.9, 10.2)	<0.001	8.0 (7.9, 8.1)	<0.001	2.1 (1.9, 2.3)	<0.001
A**ge groups**						
<60 years	4.4 (4.3, 4.5)	Ref.	8.4 (8.3, 8.5)	Ref.	0.7 (0.5, 0.9)	Ref.
60–69 years	9.4 (9.2, 9.7)	<0.001	8.9 (8.8, 9.0)	<0.001	1.8 (1.5, 2.1)	<0.001
≥70 years	20.3 (19.9, 20.8)	<0.001	9.0 (8.9, 9.1)	<0.001	3.8 (3.5, 4.2)	<0.001
**Sex**						
Male	9.1 (8.9, 9.3)	Ref.	9.1 (9.0, 9.2)	Ref.	3.3 (3.0, 3.6)	Ref.
Female	8.1 (7.9, 8.2)	<0.001	8.6 (8.5, 8.7)	<0.001	1.6 (1.4, 1.8)	<0.001
**Health insurance scheme**^b^						
UEBMI	9.0 (8.8, 9.3)	Ref.	9.5 (9.4, 9.6)	Ref.	2.3 (2.0, 2.6)	Ref.
URBMI or NRCMS	8.7 (8.6, 8.9)	0.076	8.3 (8.2, 8.4)	<0.001	2.4 (2.1, 2.8)	0.641

CI, confidence interval; UEBMI: urban employee basic medical insurance; URBMI: urban resident basic medical insurance; NRCMS: new rural cooperative medical scheme.

Data were adjusted for annually updated age, sex, study area, and the year of the index hospital admission where appropriate. Age was updated at the start of each year from 2009 to 2017 in the analysis of hospitalization rate or until the occurrence of the index hospitalization in the analysis of length of hospital stay and 30-day case fatality rate.

a We classified the study areas in Harbin, Qingdao, Gansu, and Henan as northern China and the other areas (Haikou, Suzhou, Liuzhou, Sichuan, Hunan, and Zhejiang) as southern China according to the Qinling Mountain-Huaihe River Line.

b Information on health insurance scheme is for the year of the index hospital admission. The uninsured participants were excluded from the analysis due to the small number of cases.

### Population variation in pneumonia hospitalization

The hospitalization rate and 30-day CFR increased sharply with age, peaking in the 60 and older age groups (Supplementary Figure 7). Participants aged 70 years and older had a hospitalization rate of 20·3 (95% CI: 19·9, 20·8) admissions per 1000 person-years and a 30-day CFR of 3·8 (3·5, 4·2) deaths per 100 admissions, about five times as high as those aged less than 60 years (4·4 [4·3, 4·5] admissions per 1000 person-years and 0·7 [0·5, 0·9] deaths per 100 admissions) (all *P <* 0·001) (Table 2). The differences in the mean LOS between age groups were relatively minor.

Males had a higher hospitalization rate, longer mean LOS, and higher 30-day CFR than females (all *P* < 0·001) (Table 2). Participants who enrolled in UEBMI had a longer mean LOS (*P* < 0·001) than those enrolled in other HI schemes, but there was no obvious difference in hospitalization rates and 30-day CFRs between HI schemes (*P* > 0·05).

**Figure 2:**
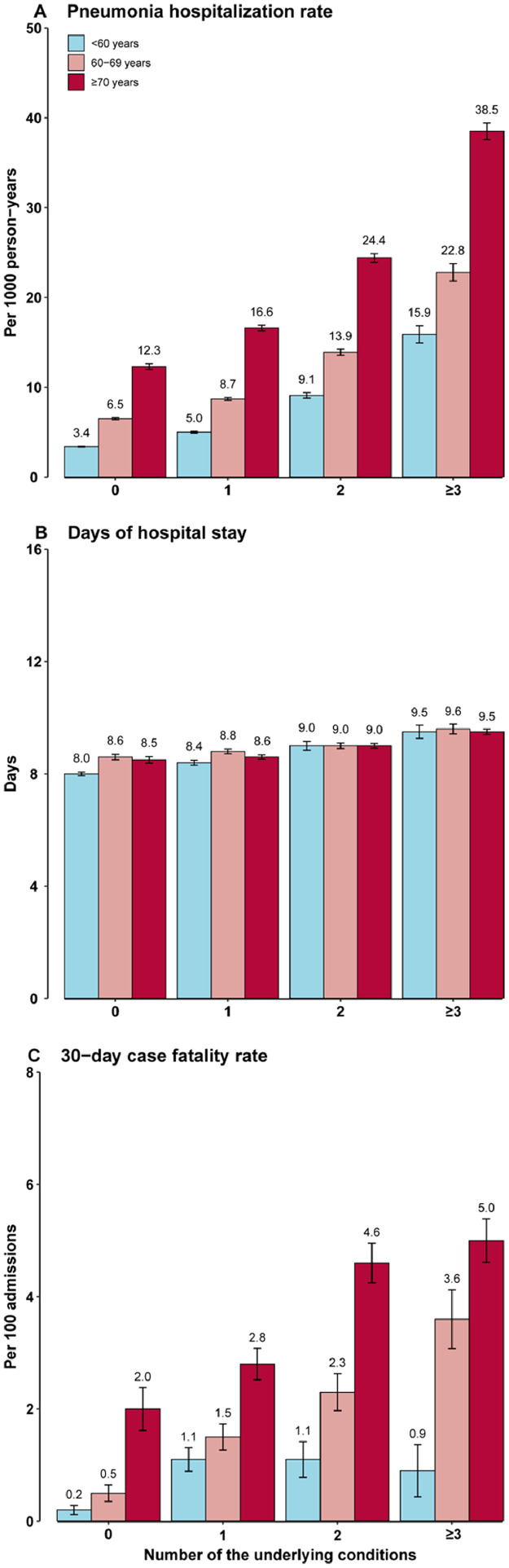
**Variations in pneumonia hospitalization by age and the number of underlying conditions**. Data were adjusted for sex, study area, and the year of the index hospital admission. Underlying conditions included hypertension, diabetes, ischemic heart disease, stroke, chronic obstructive pulmonary disease, tuberculosis, asthma, chronic kidney disease, cirrhosis/chronic hepatitis, and cancer. Age and the disease status of the above diseases were updated at the start of each year from 2009 to 2017 in the analysis of hospitalization rate or until the occurrence of the index hospitalization in the analysis of length of hospital stay and 30-day case fatality rate. The error bar represents the standard error. Numbers above the histograms are rates per 1000 person-years, mean length of stay in days, or case fatality rates per 100 admissions, as appropriate.

Compared to participants living free of concerning underlying conditions, those with any conditions (except hypertension) had higher pneumonia hospitalization rates, especially those with cancer or chronic lung diseases (all *P <* 0·05) (Table 3). The 30-day CFR was significantly higher among participants with cancer or stroke (both *P <* 0·001). Compared with participants without any underlying conditions, those with two or more conditions had a much higher hospitalization rate and 30-day CFR (all *P <* 0·001). The hospitalization rates for those having one, two, and three or more conditions were 7·8 (95% CI: 7·6, 8·0), 12·0 (11·6, 12·3), and 18·8 (18·0, 19·6) per 1000 person-years, respectively. The respective 30-day CFRs were 2·0 (1·7, 2·3), 2·9 (2·5, 3·3), and 3·4 (3·0, 3·9) deaths per 100 admissions. These findings were robust in sensitivity analyses (Supplementary Tables 2–4). Further joint analysis of age and number of underlying conditions showed the pneumonia hospitalization rate and 30-day CFR significantly and independently increased with both factors (Figure 2; all *P*_trend_ < 0·001).

**Table 3:** Variations in pneumonia hospitalization by preexisting underlying conditions.

	Pneumonia hospitalization rates per 1000 person-years		Days of hospital stay		Case-fatality rates per 100 admissions	
	Adjusted rates (95% CIs)	*P*-value	Adjusted days (95% CIs)	*P*-value	Adjusted rates (95% CIs)	*P*-value
Living free of concerning underlying conditions	5.9 (5.7, 6.0)	Ref.	8.3 (8.2, 8.4)	Ref.	0.9 (0.7, 1.2)	Ref.
Having hypertension alone	5.9 (5.7, 6.1)	0.861	8.4 (8.3, 8.5)	0.483	1.2 (0.9, 1.6)	0.145
Having diabetes alone	8.1 (7.3, 8.8)	<0.001	8.5 (8.1, 8.9)	0.318	2.1 (0.7, 3.4)	0.024
Having ischemic heart disease alone	10.8 (9.9, 11.7)	<0.001	8.9 (8.5, 9.3)	0.002	1.0 (0.3, 1.8)	0.767
Having stroke alone	10.9 (9.8, 12.0)	<0.001	9.0 (8.6, 9.4)	0.001	3.0 (1.6, 4.4)	<0.001
Having COPD alone	12.4 (11.7, 13.1)	<0.001	8.5 (8.4, 8.7)	0.044	1.4 (0.8, 2.0)	0.090
Having tuberculosis alone	12.3 (10.7, 14.0)	<0.001	9.2 (8.6, 9.8)	0.002	1.9 (0.3, 3.6)	0.113
Having asthma alone	11.1 (8.3, 13.9)	<0.001	7.5 (6.8, 8.2)	0.038	- a	- a
Having chronic kidney disease alone	8.2 (7.0, 9.4)	<0.001	8.7 (8.1, 9.4)	0.247	- a	- a
Having cirrhosis/chronic hepatitis alone	7.1 (6.0, 8.2)	0.017	8.4 (7.8, 9.0)	0.853	- a	- a
Having cancer alone	17.8 (15.7, 19.9)	<0.001	9.3 (8.8, 9.8)	<0.001	10.0 (7.4, 12.6)	<0.001
Having two underlying conditions	12.0 (11.6, 12.3)	<0.001	9.0 (8.9, 9.1)	<0.001	2.9 (2.5, 3.3)	<0.001
Having three or more underlying conditions	18.8 (18.0, 19.6)	<0.001	9.5 (9.3, 9.6)	<0.001	3.4 (3.0, 3.9)	<0.001

CI, confidence interval; COPD, chronic obstructive pulmonary disease.

Data were adjusted for annually updated age, sex, study area, and the year of the index hospital admission where appropriate.

Age and the disease status of the above diseases were updated at the start of each year from 2009 to 2017 in the analysis of hospitalization rate or until the occurrence of the index hospitalization in the analysis of length of hospital stay and 30-day case fatality rate.

a Estimates of 30-day case fatality rate were not available due to the small number of mortality cases.

## Discussion

In the present large populational-based prospective cohort study of middle-aged and older Chinese adults, we observed a gradually increasing trend in pneumonia hospitalization rate from 2009 to 2017, even after adjusting the ageing of participants and the growing burden of underlying conditions. The apparent decrease in the LOS for pneumonia mainly happened in urban residents and those enrolled in UEBMI scheme, compared with their counterparts. The 30-day CFR remained unchanged. Advanced age, male gender, and preexisting underlying conditions were associated with a higher incidence and CFR of pneumonia hospitalization.

### Comparison with other studies

Early studies in European and the US populations reported an increasing trend of pneumonia hospitalization over time among adults,^6^^,^^10^, ^11^, ^12^, ^13^ with population ageing and the increasing prevalence of underlying conditions as possible explanations. A study based on the whole Danish population found that the all-cause pneumonia hospitalization rate per 1000 person-years among adults aged 41–64, 65–79, and ≥80 years increased from 2·92, 14·06, and 31·71 in 1997 to 5·27, 21·32, and 60·38 in 2011, respectively.^12^ During the same period, the proportion of patients hospitalized with pneumonia who also suffered from underlying conditions increased from 42·9% to 46·2%.^12^ A US study using data from the National Hospital Discharge Survey showed a 20% increase in pneumonia hospitalization rate among adults aged ≥65 years from 1988 to 2002, while the proportion of pneumonia patients with chronic cardiac disease, chronic pulmonary disease, or diabetes increased from 66% to 77%.^6^

The 7-valent pneumococcal conjugate vaccine (PCV-7) was introduced into the US childhood immunization schedule in 2000. An analysis based on the National Inpatient Sample, compared the average annual pneumonia hospitalization in pre-PCV7 years (1997–1999) with late PCV7 years (2007–2009),^25^ observed a significant reduction in pneumonia hospitalization rate among children, and also seen a decline among adults, particularly pronounced among those aged ≥85 years, with hospitalization rates (per 100,000 adults) decreasing from 5697 to 4396. The reduced transmission of vaccine serotypes may contribute to the decline in the pneumonia hospitalization rate among adults.^25^ However, similar differences were not observed in studies conducted in Australian^26^ and Swedish populations.^13^ Analyses of the US Medicare data showed that the 30-day CFR for adults aged ≥65 years hospitalized for CAP decreased from 13·5% in 1987 to 9·7% in 2005, a 28·1% reduction after adjusted for age and gender.^27^ The authors discussed that the increased guideline-compliant antibiotic use and vaccination might collectively explain a large portion of the observed reduction. Another US study made a comparison of late PCV7 (2007–2009) with pre-PCV7 (1997–1999) years, which reported that there was no change in in-hospital CFR among patients hospitalized with pneumonia aged 18–64 years, but a slight decline among older patients aged ≥75 years.^25^ Similar results were observed in several studies among European adult populations that were conducted in the last 20 years.^10^^,^^12^^,^^13^

In China, neither pneumococcal nor influenza vaccines have been included in the national immunisation programme, and thus their vaccination rates among Chinese adults and children^28^^,^^29^ remained far below that in the Western populations.^30^ Thus, the secular increase in hospitalization rates for pneumonia observed in the CKB population is less likely to be affected by vaccination and is mainly attributable to increasing age and the prevalence of underlying conditions. After controlling for these two factors as well as lifestyle factors such as smoking, the remaining annual increase of 14·3% might be explained by other contributing factors such as emerging pathogens^31^ and exposure to environmental risk factors.^32^

Compared with previous studies conducted in Western populations in the mid-2010s, the CKB population showed similar hospitalization rates for pneumonia^4^^,^^33^ but relatively lower CFR.^11^^,^^33^ Using data of the US Nationwide Inpatient Sample, the estimated age-adjusted hospitalization rates (per 100,000 adults) for pneumonia between 2012 and 2014 were 119·1, 463·1, 1187·1, 2381·6, and 4304·8 for age groups 20–44, 45–64, 65–74, 75–84 and ≥85 years old, respectively.^33^ No significant change in the in-hospital CFR for pneumonia occurred between 2001 and 2014, with in-hospital CFR of 2·9%, 5·9%, 7·9%, 9·7%, and 12·4% for the above age groups. Analysis based on the electronic health record database of Hong Kong found that the hospitalization rate and CFR of pneumonia between 2011 and 2015 were close to those of Western populations.^17^ Other contemporary studies in Japanese^34^ and South Korean populations^35^ reported similar hospitalization rates and CFRs for pneumonia to our findings in CKB population. A recent study using the Chinese Urban Basic Medical Insurance (UBMI) database of 23 provinces in 2016 reported similar results to our urban population, the incidence rates (per 1000 person-years) of CAP in adults aged 30–39, 40–49, 50–59, 60–69, 70–79, and ≥85 years were 4·95, 4·48, 5·85, 7·80, 11·90, and 14·98, respectively.^5^

Studies conducted in temperate and subtropical regions for United States,^33^ United Kingdom,^10^ and Spain^36^ reported higher pneumonia hospitalization rates in colder than warmer months. Similar seasonal pattern was also observed in the Chinese study that used UBMI data, with the incidence rate peaks in winter and spring and valleys in summer and early autumn, however, this study did not examine the seasonality by geographic regions^5^. Our study further extends the evidence by showing the difference between northern and southern China. There was a larger peak in colder months and a smaller peak in warmer months in southern China, consistent with previous studies that reported dual peaks for influenza occurring in winter and summer.^16^^,^^37^^,^^38^

Variations in hospitalization rate, LOS, and 30-day CFR were observed between urban and rural areas and between Southern and Northern China, which might be associated with the regional variations in healthcare-seeking behaviours and health insurance reimbursement policies.^23^^,^^39^ In line with previous studies,^5^, ^6^, ^7^, ^8^, ^9^^,^^17^^,^^40^^,^^9^^,^^10^^,^^12^^,^^41^ the hospitalization rate and CFR for pneumonia hospitalization in the CKB population increased with age, with a steep increase since aged 60 years old and above, and were significantly higher among participants with underlying conditions. Due to the lack of reasonable estimates of the denominator population, previous studies based on administrative claim data usually reported the proportion of pneumonia patients with underlying conditions, but were difficult to estimate the hospitalization rate for pneumonia by underlying condition types. Only a few studies compared the CFR for pneumonia by underlying conditions^42^ or comorbidity index.^41^ Analyses of the US Medicare data, including 623,718 patients aged ≥65 years hospitalized for CAP in 1997, reported that patients with congestive heart failure, malignancy, myocardial infarction, renal disease, or liver disease had a higher risk of in-hospital CFR than those without underlying condition; the odds ratios (95% CIs) were 1·53 (1·47, 1·59), 2·26 (2·20, 2·33), 1·53 (1·47, 1·59), 2·15 (1·95, 2·25), and 2·10 (1·87, 2·34), respectively.^42^ A population-based study conducted in Denmark between 1994 and 2004 found that patients with a higher comorbidity index score had a poor prognosis, with 30-day CFR of pneumonia hospitalization being 10·5%, 15·8%, and 21·0% for those who were classified as low, medium, and high level of comorbidity, respectively.^41^ Our study provided the estimates of the hospitalization rate and 30-day CFR for pneumonia hospitalization by age groups and numbers of underlying conditions, showing that adults aged <60 years with one or more underlying conditions had similar or even higher risks than those aged 60–69 years without underlying conditions. In the context of the increasing prevalence of chronic diseases at a younger age,^33^ middle-aged adults with underlying conditions should also be reminded to prevent the development of pneumonia.

### Strengths and limitations

To our knowledge, this is the first population-based study that provides detailed epidemiological features of pneumonia hospitalization in both urban and rural Chinese adults of middle age and above. The main strength of our study lies in the large sample size and the inclusion of diverse geographic, socioeconomic, and age groups of populations. The comprehensive linkage to the HI system allows us for trend analysis over a 9-year study period. An explicit denominator population and the collection of extensive individual-level data enable further analyses by subpopulations and would better inform the priorities.

Our study also has limitations. First, the CKB study represents multiple areas and socioeconomic groups rather than the whole Chinese population. Therefore, the extrapolation of the results must be done with caution. Second, we only included patients requiring hospitalization and mild cases might be missed for those who were not hospitalized. Third, we did not have information on causal pathogens of pneumonia through HI system, because the pathogen detection rate was typically low in routine clinical practice.^6^^,^^7^^,^^10^^,^^11^^,^^17^ The potential reasons for the low pathogen detection rate included the emphasis on early antibiotic initiation, the difficulty to obtain lower respiratory tract specimens, the insensitive diagnostic tests for known pathogens, or the existence of unidentified pathogens.^7^ Fourth, we were unable to distinguish CAP from hospital-acquired pneumonia according to the information from HI data. However, our findings were generally unchanged when restricted analyses in participants without hospital admission in the previous 30 days. Fifth, the lack of sufficient information limited us from conducting an in-depth analysis to obtain direct evidence to explain the long-term increasing trend, as well as the unusual fluctuations in individual years of pneumonia hospitalization rate.

Based on a large population-based cohort study of the Chinese population, we found a substantial and increasing hospitalization burden of pneumonia among middle-aged and older Chinese adults from 2009 to 2017. There were important differences in the incidence and CFR of adults hospitalized with pneumonia across regions and subpopulations of different ages and underlying conditions. The recommendation of proven prevention measures should be reinforced, particularly in high-risk groups, considering the comparatively higher healthcare cost and the poorer outcome. Besides, our study also provided parameters to help determine needs for preventive measures and medical resources and parameters for health economics assessments.

## Contributors

JL conceived and designed the paper. LL, ZC, and JC, as the members of CKB steering committee, designed and supervised the conduct of the whole study, obtained funding, and together with CY, YG, PP, LY, YC, HD, NW, S.B., A.H. and DS acquired the data. YHu and YHan analysed the data. YHu drafted the manuscript. JL, LL, and YP contributed to the interpretation of the results. JL critically reviewed and revised the manuscript for important intellectual content. All authors reviewed and approved the final manuscript. JL is the guarantor. All authors had access to all data and responsibility for the decision to submit for publication.

## Declaration of interests

We declare that we have no conflict of interest.

## Appendix Supplementary materials

Image, application 1

Image, application 2
