# Supplementary material for: The hospitalization burden of all-cause pneumonia in China: A population-based study, 2009–2017
Source: Lancet Reg Health West Pac. 2022 Apr 6;22:100443. doi: 10.1016/j.lanwpc.2022.100443 (PMC8991381; doi:10.1016/j.lanwpc.2022.100443)
Supplement: Supplementary file 1 [file mmc1.docx]

**Table of Contents**

**Page 2:** Members of the China Kadoorie Biobank collaborative group

Page 3: Supplementary Table 1. Background characteristics of participants by the number of pneumonia hospitalizations during 2009-2017

**Page 5:** **Supplementary Table 2.** Regional and population variations in pneumonia hospitalization after full adjustment for potential confounders

Page 7: Supplementary Table 3. Regional and population variations in pneumonia hospitalization by restricting analysis in the first-ever hospital admission for pneumonia

Page 8: Supplementary Table 4. Regional and population variations in pneumonia hospitalization by restricting analysis in participants without any other hospital admission in the previous 30 days

Page 10: Supplementary Figure 1. Comparisons between different analysis strategies for secular trends in pneumonia hospitalization

**Page 11:** **Supplementary Figure 2.** Regional variation in secular trends of pneumonia hospitalization from 2009 to 2017

**Page 12: Supplementary Figure 3.** Population variation in secular trends of pneumonia hospitalization from 2009 to 2017

Page 14: Supplementary Figure 4. Sensitivity analyses for secular trend by restricting analysis in the first-ever hospital admission for pneumonia

**Page 15: Supplementary Figure 5.** Sensitivity analyses for secular trend by restricting analysis in participants without any other hospital admission in the previous 30 days

**Page 16: Supplementary Figure 6.** Seasonal variation in pneumonia hospitalization from 2009 to 2017

**Page 17: Supplementary Figure 7.** Variations in pneumonia hospitalization by age groups

**Members of the China Kadoorie Biobank collaborative group**

**International Steering Committee:** Junshi Chen, Zhengming Chen (PI), Robert Clarke, Rory Collins, Yu Guo, Liming Li (PI), Jun Lv, Richard Peto, Robin Walters. **International Co-ordinating Centre, Oxford:** Daniel Avery, Ruth Boxall, Derrick Bennett, Yumei Chang, Yiping Chen, Zhengming Chen, Robert Clarke, Huaidong Du, Simon Gilbert, Alex Hacker, Mike Hill, Michael Holmes, Andri Iona, Christiana Kartsonaki, Rene Kerosi, Ling Kong, Om Kurmi, Garry Lancaster, Sarah Lewington, Kuang Lin, John McDonnell, Iona Millwood, Qunhua Nie, Jayakrishnan Radhakrishnan, Paul Ryder, Sam Sansome, Dan Schmidt, Paul Sherliker, Rajani Sohoni, Becky Stevens, Iain Turnbull, Robin Walters, Jenny Wang, Lin Wang, Neil Wright, Ling Yang, Xiaoming Yang. **National Co-ordinating Centre, Beijing:** Yu Guo, Xiao Han, Can Hou, Jun Lv, Pei Pei, Chao Liu, Canqing Yu. **10 Regional Co-ordinating Centres: Qingdao CDC:** Zengchang Pang, Ruqin Gao, Shanpeng Li, Shaojie Wang, Yongmei Liu, Ranran Du, Yajing Zang, Liang Cheng, Xiaocao Tian, Hua Zhang, Yaoming Zhai, Feng Ning, Xiaohui Sun, Feifei Li. **Licang CDC:** Silu Lv, Junzheng Wang, Wei Hou. **Heilongjiang Provincial CDC:** Mingyuan Zeng, Ge Jiang, Xue Zhou. **Nangang CDC:** Liqiu Yang, Hui He, Bo Yu, Yanjie Li, Qinai Xu,Quan Kang, Ziyan Guo. **Hainan Provincial CDC:** Dan Wang, Ximin Hu, Jinyan Chen, Yan Fu, Zhenwang Fu, Xiaohuan Wang. **Meilan CDC:** Min Weng, Zhendong Guo, Shukuan Wu,Yilei Li, Huimei Li, Zhifang Fu. **Jiangsu Provincial CDC:** Ming Wu, Yonglin Zhou, Jinyi Zhou, Ran Tao, Jie Yang, Jian Su. **Suzhou CDC:** Fang liu, Jun Zhang, Yihe Hu, Yan Lu, , Liangcai Ma, Aiyu Tang, Shuo Zhang, Jianrong Jin, Jingchao Liu. **Guangxi Provincial CDC:** Zhenzhu Tang, Naying Chen, Ying Huang. **Liuzhou CDC:** Mingqiang Li, Jinhuai Meng, Rong Pan, Qilian Jiang, Jian Lan,Yun Liu, Liuping Wei, Liyuan Zhou, Ningyu Chen Ping Wang, Fanwen Meng, Yulu Qin,, Sisi Wang. **Sichuan Provincial CDC:** Xianping Wu, Ningmei Zhang, Xiaofang Chen,Weiwei Zhou. **Pengzhou CDC:** Guojin Luo, Jianguo Li, Xiaofang Chen, Xunfu Zhong, Jiaqiu Liu, Qiang Sun. **Gansu Provincial CDC:** Pengfei Ge, Xiaolan Ren, Caixia Dong. **Maiji CDC:** Hui Zhang, Enke Mao, Xiaoping Wang, Tao Wang, Xi zhang. **Henan Provincial CDC:** Ding Zhang, Gang Zhou, Shixian Feng, Liang Chang, Lei Fan. **Huixian CDC:** Yulian Gao, Tianyou He, Huarong Sun, Pan He, Chen Hu, Xukui Zhang, Huifang Wu, Pan He. **Zhejiang Provincial CDC:** Min Yu, Ruying Hu, Hao Wang. Tongxiang CDC: Yijian Qian, Chunmei Wang, Kaixu Xie, Lingli Chen, Yidan Zhang, Dongxia Pan, Qijun Gu. **Hunan Provincial CDC:** Yuelong Huang, Biyun Chen, Li Yin, Huilin Liu, Zhongxi Fu, Qiaohua Xu. **Liuyang CDC:** Xin Xu, Hao Zhang, Huajun Long, Xianzhi Li, Libo Zhang, Zhe Qiu.

Supplementary Table 1. Background characteristics of participants by the number of pneumonia hospitalizations during 2009-2017

|  | **Number of pneumonia hospitalizations** | | | |
| --- | --- | --- | --- | --- |
|  | **0 (n=478 207)** | **1 (n=22 226)** | **2 (n=3 961)** | **≥3 (1 692)** |
| **Age at the beginning of 2009, year (SD)** | 53.4 (10.5) | 59.2 (10.6) | 61.3 (10.1) | 62.2 (9.8) |
| **Age groups at the beginning of 2009, n (%)** |  |  |  |  |
| <60 years | 71.7 | 50.2 | 41.5 | 38.3 |
| 60-69 years | 19.2 | 28.4 | 32.4 | 33.1 |
| ≥70 years | 9.1 | 21.4 | 26.1 | 28.6 |
| **Sex, n (%)** |  |  |  |  |
| Male | 40.7 | 40.6 | 42.9 | 48.5 |
| Female | 59.3 | 59.4 | 57.1 | 51.5 |
| **Region, n (%)** |  |  |  |  |
| Urban | 44.6 | 38.6 | 36.4 | 40.2 |
| Rural | 55.4 | 61.4 | 63.6 | 59.8 |
| **Geographic location ^a^, n (%)** |  |  |  |  |
| Northern China | 40.8 | 30.2 | 24.6 | 24.9 |
| Southern China | 59.2 | 69.8 | 75.4 | 75.1 |
| **Education, n (%)** |  |  |  |  |
| No formal school | 18.3 | 18.9 | 19.4 | 19.2 |
| Primary or middle school | 60.5 | 60.9 | 61.1 | 59.8 |
| High school or above | 21.1 | 20.2 | 19.5 | 21.0 |
| **Health insurance scheme ^b^, n (%)** |  |  |  |  |
| UEBMI | 37.5 | 39.4 | 40.2 | 42.2 |
| URBMI or NRCMS | 56.8 | 57.6 | 57.9 | 56.0 |
| Uninsured | 5.7 | 2.9 | 1.9 | 1.8 |
| **Physical activity, MET-h/day (SD)** | 21.2 (13.9) | 21.0 (12.6) | 21.2 (12.2) | 20.6 (12.1) |
| **Tobacco smoking ^c^, n (%)** |  |  |  |  |
| Non-current daily smoker | 70.9 | 69.0 | 68.3 | 68.6 |
| 1-14 cig/d | 10.8 | 10.7 | 11.3 | 10.6 |
| 15-24 cig/d | 13.2 | 14.4 | 14.2 | 14.2 |
| ≥25 cig/d | 5.1 | 5.9 | 6.2 | 6.5 |
| **Alcohol drinking, n (%)** |  |  |  |  |
| Non-current daily drinker | 91.0 | 91.2 | 91.7 | 92.4 |
| Daily male<30/female<15g pure alcohol | 2.1 | 2.0 | 1.8 | 1.5 |
| Daily male≥30/female≥15g pure alcohol | 6.9 | 6.8 | 6.4 | 6.2 |
| **Body-mass index ^d^, n (%)** |  |  |  |  |
| Underweight, <18.5 kg/m^2^ | 4.1 | 5.7 | 6.5 | 6.6 |
| Normal, 18.5-23.9 kg/m^2^ | 51.9 | 51.7 | 51.2 | 51.5 |
| Overweight or obesity, ≥24.0 kg/m^2^ | 44.0 | 42.7 | 42.3 | 41.8 |
| **Waist circumference, n (%)** |  |  |  |  |
| Male<85 cm, female<80 cm | 57.9 | 58.3 | 58.1 | 60.4 |
| Male≥85 cm, female≥80 cm | 42.1 | 41.7 | 41.9 | 39.6 |
| **Prevalence of underlying conditions at the beginning of 2009, n** **(%)** | |  |  |  |
| Hypertension | 35.6 | 35.0 | 34.0 | 31.4 |
| Diabetes | 6.2 | 6.8 | 7.1 | 6.8 |
| Ischemic heart disease | 4.0 | 5.1 | 5.7 | 7.0 |
| Stroke | 2.6 | 3.0 | 3.0 | 3.5 |
| COPD | 6.9 | 10.7 | 12.4 | 13.8 |
| Tuberculosis | 1.5 | 2.2 | 2.7 | 3.8 |
| Asthma | 0.6 | 1.0 | 1.5 | 2.1 |
| Chronic kidney disease | 1.5 | 1.9 | 1.8 | 2.1 |
| Cirrhosis/chronic hepatitis | 1.5 | 1.6 | 1.6 | 1.8 |
| Cancer | 1.0 | 1.2 | 1.5 | 2.3 |
| **Number of the above conditions at the beginning of 2009, n** **(%)** |  |  |  |  |
| 0 | 53.7 | 49.8 | 48.7 | 46.8 |
| 1 | 34.1 | 35.0 | 34.3 | 34.7 |
| 2 | 9.6 | 11.7 | 13.1 | 13.8 |
| ≥3 | 2.5 | 3.4 | 3.9 | 4.8 |

SD: standard deviation; UEBMI: urban employee basic medical insurance; URBMI: urban resident basic medical insurance; NRCMS: new rural cooperative medical scheme; MET: metabolic equivalent task; COPD: chronic obstructive pulmonary disease.

All variables were measured at baseline unless indicated otherwise.

Data were adjusted for age at corresponding year, sex, and study area where appropriate.

^a^ We classified the study areas in Harbin, Qingdao, Gansu, and Henan as northern China and the other areas including Haikou, Suzhou, Liuzhou, Sichuan, Hunan, and Zhejiang as southern China according to the Qinling Mountain-Huaihe River Line.

^b^ Information on health insurance scheme is for the year 2012 (n=494,511).

^c^ Participants who quit smoking because of illness were classified as current daily smokers.

^d^ Two participants had missing data on the body-mass index.

**Supplementary Table 2. Regional and population variations in pneumonia hospitalization after full adjustment for potential confounders**

|  | **Pneumonia hospitalization rates per 1000 person-years** | |  | **Days of hospital stay** | |  | **Case-fatality rates per 100 admissions** | |
| --- | --- | --- | --- | --- | --- | --- | --- | --- |
|  | **Fully adjusted rates (95% CIs)** | ***P*-value** |  | **Fully adjusted days (95% CIs)** | ***P*-value** |  | **Fully adjusted rates (95% CIs)** | ***P*-value** |
| **Region** |  |  |  |  |  |  |  |  |
| Urban | 7.5 (7.3, 7.7) | Ref. |  | 10.0 (9.9, 10.1) | Ref. |  | 2.6 (2.3, 2.9) | Ref. |
| Rural | 9.6 (9.3, 9.8) | <0.001 |  | 7.9 (7.8, 8.0) | <0.001 |  | 2.1 (1.8, 2.4) | 0.031 |
| **Geographic location** **^a^** |  |  |  |  |  |  |  |  |
| Northern China | 5.9 (5.8, 6.1) | Ref. |  | 10.5 (10.4, 10.6) | Ref. |  | 2.9 (2.5, 3.3) | Ref. |
| Southern China | 10.3 (10.1, 10.4) | <0.001 |  | 8.2 (8.1, 8.2) | <0.001 |  | 2.1 (1.9, 2.4) | 0.001 |
| **Age groups** |  |  |  |  |  |  |  |  |
| <60 years | 5.2 (5.0, 5.3) | Ref. |  | 8.5 (8.4, 8.6) | Ref. |  | 0.9 (0.6, 1.1) | Ref. |
| 60-69 years | 8.8 (8.6, 9.1) | <0.001 |  | 9.0 (8.9, 9.1) | <0.001 |  | 1.8 (1.5, 2.0) | <0.001 |
| ≥70 years | 15.1 (14.7, 15.5) | <0.001 |  | 8.9 (8.8, 9.0) | <0.001 |  | 3.5 (3.1, 3.8) | <0.001 |
| **Sex** |  |  |  |  |  |  |  |  |
| Male | 8.9 (8.7, 9.1) | Ref. |  | 9.0 (8.9, 9.1) | Ref. |  | 3.1 (2.8, 3.5) | Ref. |
| Female | 8.4 (8.2, 8.6) | 0.004 |  | 8.6 (8.6, 8.7) | <0.001 |  | 1.6 (1.4, 1.9) | <0.001 |
| **Health insurance scheme ^b^** |  |  |  |  |  |  |  |  |
| UEBMI | 9.1 (8.8, 9.4) | Ref. |  | 9.4 (9.3, 9.5) | Ref. |  | 2.3 (2.0, 2.6) | Ref. |
| URBMI or NRCMS | 8.9 (8.8, 9.1) | 0.333 |  | 8.3 (8.3, 8.4) | <0.001 |  | 2.4 (2.1, 2.8) | 0.556 |
| **Number** **of the underlying conditions ^c^** | |  |  |  |  |  |  |  |
| 0 | 5.8 (5.7, 5.9) | Ref. |  | 8.3 (8.2, 8.4) | Ref. |  | 0.9 (0.6, 1.2) | Ref. |
| 1 | 7.9 (7.7, 8.0) | <0.001 |  | 8.6 (8.5, 8.7) | <0.001 |  | 1.9 (1.6, 2.2) | <0.001 |
| 2 | 12.1 (11.7, 12.4) | <0.001 |  | 9.0 (8.9, 9.1) | <0.001 |  | 2.9 (2.5, 3.3) | <0.001 |
| ≥3 | 18.9 (18.1, 19.7) | <0.001 |  | 9.4 (9.2, 9.6) | <0.001 |  | 3.5 (3.0, 4.0) | <0.001 |

CI, confidence interval; UEBMI: urban employee basic medical insurance; URBMI: urban resident basic medical insurance; NRCMS: new rural cooperative medical scheme.

The data were adjusted for annually updated age, sex, study area, education, health insurance scheme, physical activity, tobacco smoking, alcohol consumption, body mass index, waist circumference, the year of the index hospital admission, and the prevalence of hypertension, diabetes, ischemic heart disease, stroke, chronic obstructive pulmonary disease, tuberculosis, asthma, chronic kidney disease, cirrhosis/chronic hepatitis, and cancer where appropriate. Age and the disease status of the above diseases were updated at the start of each year from 2009 to 2017 in the analysis of hospitalization rate or until the occurrence of the index hospitalization in the analysis of length of hospital stay and 30-day case fatality rate.

^a^ We classified the study areas in Harbin, Qingdao, Gansu, and Henan as northern China and the other areas (Haikou, Suzhou, Liuzhou, Sichuan, Hunan, and Zhejiang) as southern China according to the Qinling Mountain-Huaihe River Line.

^b^ Information on health insurance scheme is for the year of the index hospital admission. The uninsured participants were excluded from the analysis due to the small number of cases.

^c^ Underlying conditions included hypertension, diabetes, ischemic heart disease, stroke, chronic obstructive pulmonary disease, tuberculosis, asthma, chronic kidney disease, cirrhosis/chronic hepatitis, and cancer.

Supplementary Table 3. Regional and population variations in pneumonia hospitalization by restricting analysis in the first-ever hospital admission for pneumonia

|  | **Pneumonia hospitalization rates per 1000 person-years** | | | |  | **Days of hospital stay** | | |
| --- | --- | --- | --- | --- | --- | --- | --- | --- |
|  | **Adjusted rates (95% CIs)** | ***P*-value** | | |  | **Adjusted days (95% CIs)** | | ***P*-value** |
| **Region** |  |  |  |  | | |  | |
| Urban | 5.5 (5.4, 5.6) | Ref. |  | 10.4 (10.3, 10.5) | | | Ref. | |
| Rural | 7.2 (7.1, 7.3) | <0.001 |  | 7.4 (7.3, 7.5) | | | <0.001 | |
| **Geographic location ^a^** |  |  |  |  | | |  | |
| Northern China | 4.7 (4.6, 4.8) | Ref. |  | 10.8 (10.6, 10.9) | | | Ref. | |
| Southern China | 7.5 (7.4, 7.6) | <0.001 |  | 7.7 (7.6, 7.8) | | | <0.001 | |
| **Age groups** |  |  |  |  | | |  | |
| <60 years | 3.6 (3.5, 3.6) | Ref. |  | 8.2 (8.1, 8.3) | | | Ref. | |
| 60-69 years | 7.3 (7.1, 7.4) | <0.001 |  | 8.7 (8.6, 8.8) | | | <0.001 | |
| ≥70 years | 14.6 (14.3, 14.9) | <0.001 |  | 8.7 (8.7, 8.8) | | | <0.001 | |
| **Sex** |  |  |  |  | | |  | |
| Male | 6.6 (6.5, 6.8) | Ref. |  | 8.8 (8.7, 8.9) | | | Ref. | |
| Female | 6.3 (6.2, 6.4) | <0.001 |  | 8.4 (8.4, 8.5) | | | <0.001 | |
| **Health insurance scheme ^b^** |  |  |  |  | | |  | |
| UEBMI | 6.8 (6.6, 6.9) | Ref. |  | 9.3 (9.2, 9.4) | | | Ref. | |
| URBMI or NRCMS | 6.7 (6.6, 6.8) | 0.314 |  | 8.0 (8.0, 8.1) | | | <0.001 | |
| **Number** **of the underlying conditions ^c^** | |  |  |  | | |  | |
| 0 | 4.9 (4.8, 5.0) | Ref. |  | 8.2 (8.1, 8.3) | | | Ref. | |
| 1 | 6.1 (6.0, 6.3) | <0.001 |  | 8.4 (8.3, 8.5) | | | 0.004 | |
| 2 | 8.7 (8.5, 8.9) | <0.001 |  | 8.8 (8.6, 8.9) | | | <0.001 | |
| ≥3 | 11.7 (11.3, 12.1) | <0.001 |  | 9.2 (9.1, 9.4) | | | <0.001 | |

CI, confidence interval; UEBMI: urban employee basic medical insurance; URBMI: urban resident basic medical insurance; NRCMS: new rural cooperative medical scheme.

Data were adjusted for annually updated age, sex, study area, and the year of the index hospital admission where appropriate.

Age and the the number of underlying conditions were updated at the start of each year from 2009 to 2017 in the analysis of hospitalization rate or until the occurrence of the index hospitalization in the analysis of length of hospital stay and 30-day case fatality rate.

^a^ We classified the study areas in Harbin, Qingdao, Gansu, and Henan as northern China and the other areas (Haikou, Suzhou, Liuzhou, Sichuan, Hunan, and Zhejiang) as southern China according to the Qinling Mountain-Huaihe River Line.

^b^ Information on health insurance scheme is for the year of the index hospital admission. The uninsured participants were excluded from the analysis due to the small number of cases.

^c^ Underlying conditions included hypertension, diabetes, ischemic heart disease, stroke, chronic obstructive pulmonary disease, tuberculosis, asthma, chronic kidney disease, cirrhosis/chronic hepatitis, and cancer.

Supplementary Table 4. Regional and population variations in pneumonia hospitalization by restricting analysis in participants without any other hospital admission in the previous 30 days

|  | **Pneumonia hospitalization rates per 1000 person-years** | |  | **Days of hospital stay** | |  | **Case-fatality rates per 100 admissions** | |
| --- | --- | --- | --- | --- | --- | --- | --- | --- |
|  | **Adjusted rates (95% CIs)** | ***P*-value** |  | **Adjusted days (95% CIs)** | ***P*-value** |  | **Adjusted rates (95% CIs)** | ***P*-value** |
| **Region** |  |  |  |  |  |  |  |  |
| Urban | 6.4 (6.3, 6.6) | Ref. |  | 10.5 (10.4, 10.6) | Ref. |  | 2.4 (2.1, 2.7) | Ref. |
| Rural | 8.4 (8.2, 8.5) | <0.001 |  | 7.4 (7.3, 7.4) | <0.001 |  | 1.6 (1.4, 1.8) | 0.001 |
| **Geographic location ^a^** |  |  |  |  |  |  |  |  |
| Northern China | 5.2 (5.1, 5.4) | Ref. |  | 10.9 (10.7, 11.0) | Ref. |  | 2.6 (2.3, 3.0) | Ref. |
| Southern China | 8.8 (8.7, 9.0) | <0.001 |  | 7.8 (7.7, 7.9) | <0.001 |  | 1.7 (1.5, 1.9) | <0.001 |
| **Age groups** |  |  |  |  |  |  |  |  |
| <60 years | 4.0 (3.9, 4.1) | Ref. |  | 8.2 (8.1, 8.3) | Ref. |  | 0.5 (0.3, 0.7) | Ref. |
| 60-69 years | 8.3 (8.1, 8.5) | <0.001 |  | 8.7 (8.6, 8.8) | <0.001 |  | 1.3 (1.0, 1.5) | <0.001 |
| ≥70 years | 17.5 (17.1, 17.8) | <0.001 |  | 8.8 (8.7, 8.9) | <0.001 |  | 3.3 (3.0, 3.7) | <0.001 |
| **Sex** |  |  |  |  |  |  |  |  |
| Male | 7.8 (7.6, 8.0) | Ref. |  | 8.8 (8.7, 8.9) | Ref. |  | 2.6 (2.4, 2.9) | Ref. |
| Female | 7.2 (7.1, 7.3) | <0.001 |  | 8.4 (8.4, 8.5) | <0.001 |  | 1.3 (1.1, 1.5) | <0.001 |
| **Health insurance scheme ^b^** |  |  |  |  |  |  |  |  |
| UEBMI | 8.0 (7.8, 8.2) | Ref. |  | 9.3 (9.2, 9.4) | Ref. |  | 1.9 (1.6, 2.2) | Ref. |
| URBMI or NRCMS | 7.7 (7.5, 7.8) | 0.030 |  | 8.1 (8.0, 8.2) | <0.001 |  | 2.0 (1.7, 2.4) | 0.573 |
| **Number** **of the underlying conditions ^c^** | |  |  |  |  |  |  |  |
| 0 | 5.3 (5.2, 5.4) | Ref. |  | 8.2 (8.1, 8.3) | Ref. |  | 0.9 (0.6, 1.2) | Ref. |
| 1 | 7.0 (6.8, 7.1) | <0.001 |  | 8.5 (8.4, 8.6) | 0.001 |  | 1.6 (1.3, 1.9) | 0.001 |
| 2 | 10.4 (10.1, 10.7) | <0.001 |  | 8.8 (8.6, 8.9) | <0.001 |  | 2.5 (2.1, 2.9) | <0.001 |
| ≥3 | 15.6 (14.9, 16.2) | <0.001 |  | 9.2 (9.0, 9.4) | <0.001 |  | 2.6 (2.2, 3.1) | <0.001 |

CI, confidence interval; UEBMI: urban employee basic medical insurance; URBMI: urban resident basic medical insurance; NRCMS: new rural cooperative medical scheme.

Data were adjusted for annually updated age, sex, study area, and the year of the index hospital admission where appropriate.

Age and the the number of underlying conditions were updated at the start of each year from 2009 to 2017 in the analysis of hospitalization rate or until the occurrence of the index hospitalization in the analysis of length of hospital stay and 30-day case fatality rate.

^a^ We classified the study areas in Harbin, Qingdao, Gansu, and Henan as northern China and the other areas (Haikou, Suzhou, Liuzhou, Sichuan, Hunan, and Zhejiang) as southern China according to the Qinling Mountain-Huaihe River Line.

^b^ Information on health insurance scheme is for the year of the index hospital admission. The uninsured participants were excluded from the analysis due to the small number of cases.

^c^ Underlying conditions included hypertension, diabetes, ischemic heart disease, stroke, chronic obstructive pulmonary disease, tuberculosis, asthma, chronic kidney disease, cirrhosis/chronic hepatitis, and cancer.


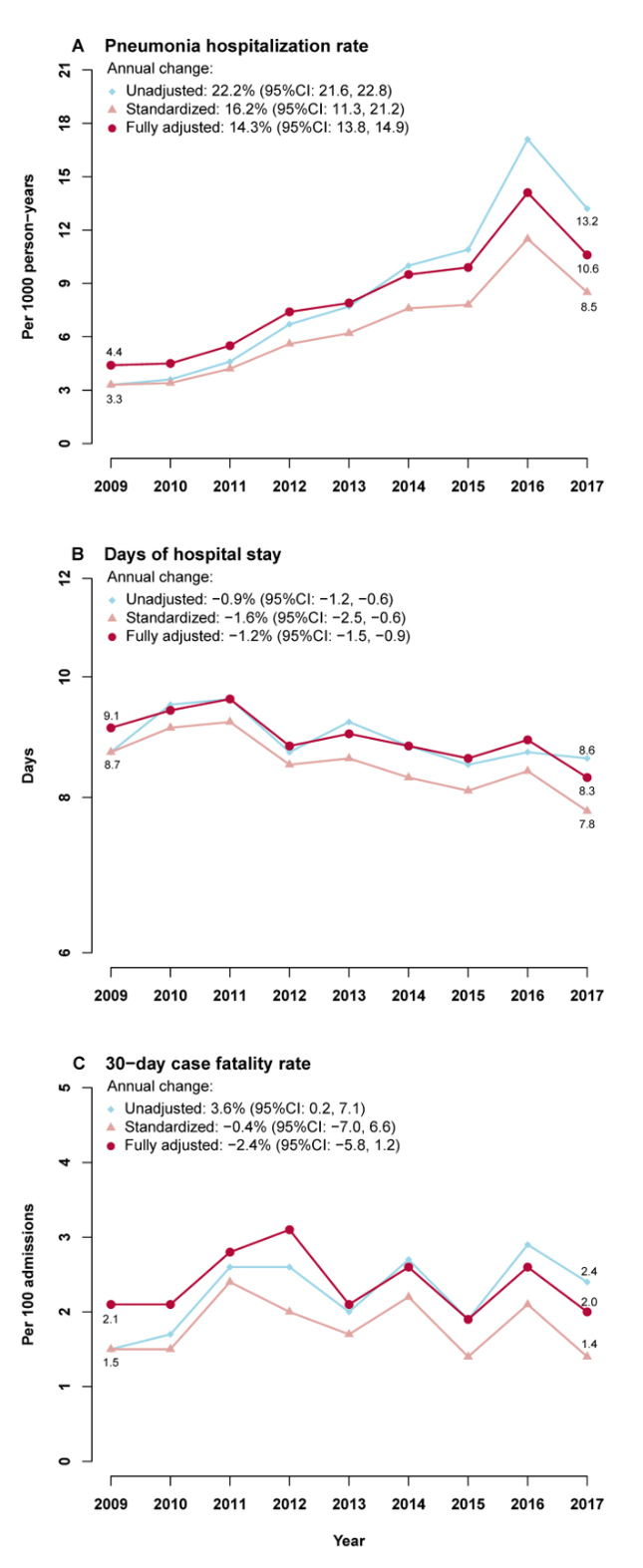


Supplementary Figure 1. Comparisons between different analysis strategies for secular trends in pneumonia hospitalization

(1) Standardized method: the standardized values presented here were standardized to the age, sex, and regional distribution of the overall CKB population in 2009. (2) Fully adjusted model: the data were adjusted for annually updated age, sex, study area, education, health insurance scheme, physical activity, tobacco smoking, alcohol consumption, body mass index, waist circumference, and the prevalence of hypertension, diabetes, ischemic heart disease, stroke, chronic obstructive pulmonary disease, tuberculosis, asthma, chronic kidney disease, cirrhosis/chronic hepatitis, and cancer. Age and the disease status of the above diseases were updated at the start of each year from 2009 to 2017 in the analysis of hospitalization rate or until the occurrence of the index hospitalization in the analysis of length of hospital stay and 30-day case fatality rate.


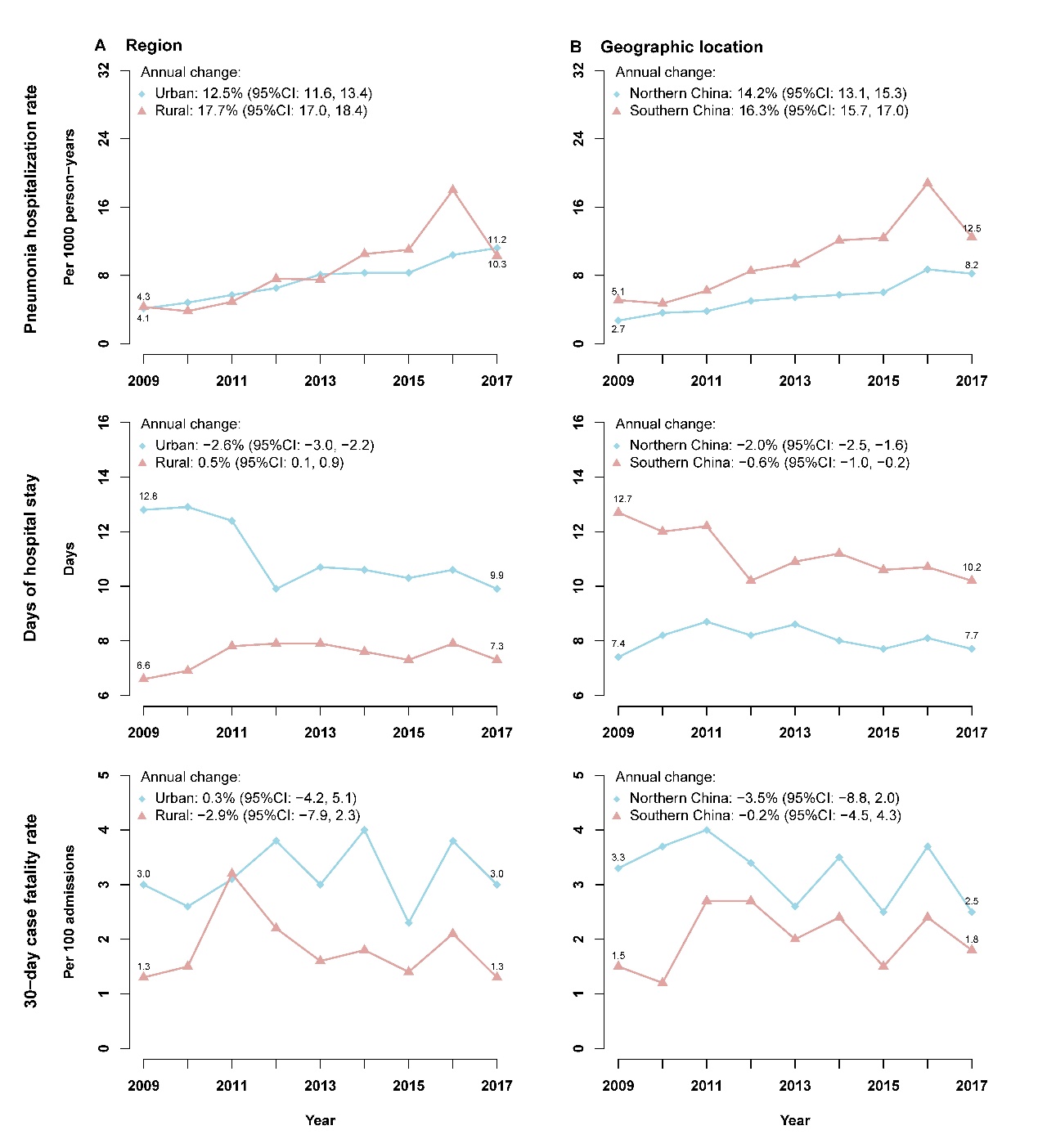


**Supplementary Figure 2. Regional variation in secular trends of pneumonia hospitalization from 2009 to 2017**

Data were adjusted for annually updated age, sex, and study area where appropriate. Age was updated at the start of each year from 2009 to 2017 in the analysis of hospitalization rate or until the occurrence of the index hospitalization in the analysis of length of hospital stay and 30-day case fatality rate. We classified the study areas in Harbin, Qingdao, Gansu, and Henan as northern China and the other areas (Haikou, Suzhou, Liuzhou, Sichuan, Hunan, and Zhejiang) as southern China according to the Qinling Mountain-Huaihe River Line.


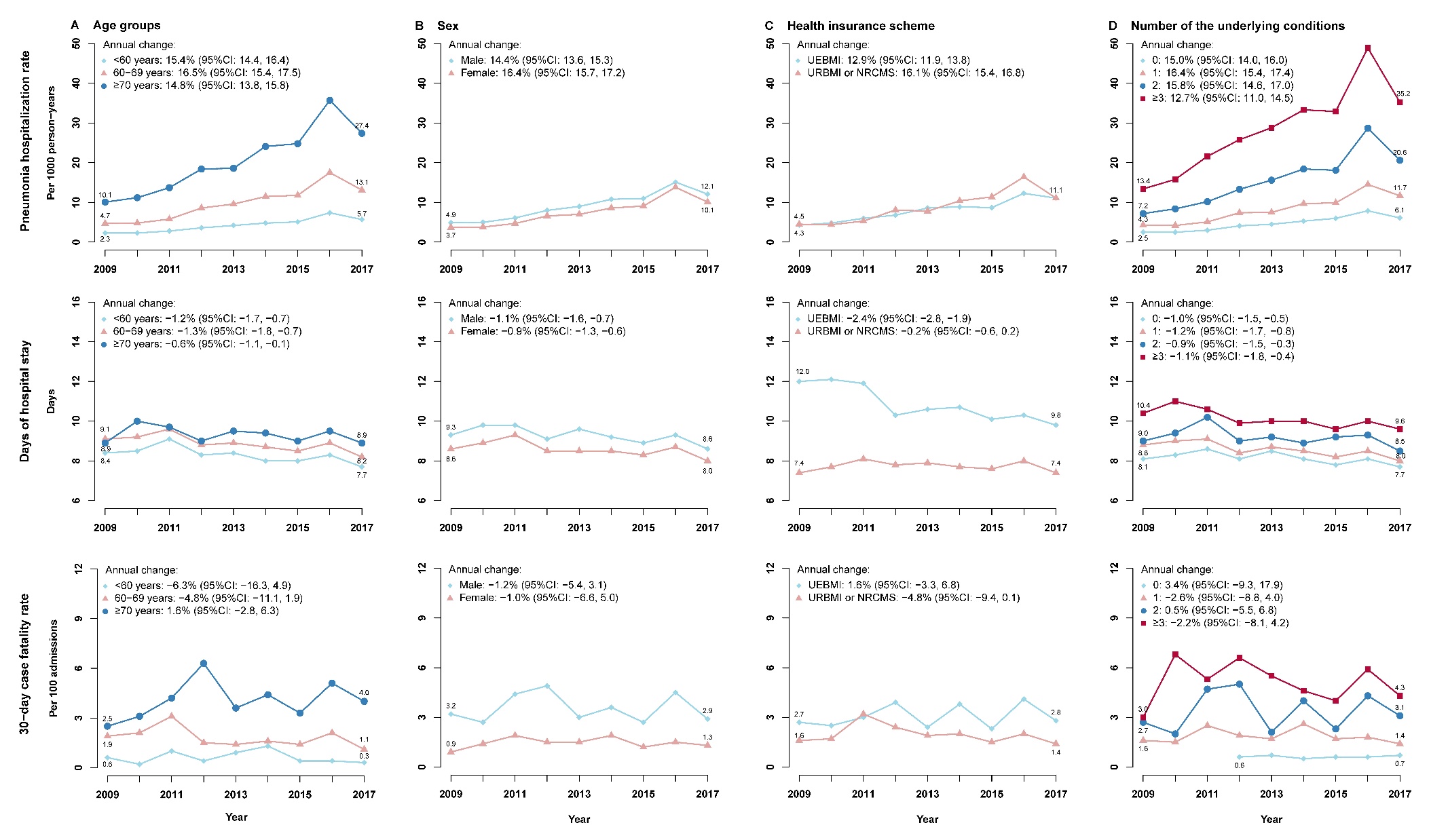


**Supplementary Figure 3. Population variation in secular trends of pneumonia hospitalization from 2009 to 2017**

UEBMI: urban employee basic medical insurance; URBMI: urban resident basic medical insurance; NRCMS: new rural cooperative medical scheme.

Data were adjusted for annually updated age, sex, and study area where appropriate.

Underlying conditions included hypertension, diabetes, ischemic heart disease, stroke, chronic obstructive pulmonary disease, tuberculosis, asthma, chronic kidney disease, cirrhosis/chronic hepatitis, and cancer. Age and the disease status of the above diseases were updated at the start of each year from 2009 to 2017 in the analysis of hospitalization rate or until the occurrence of the index hospitalization in the analysis of length of hospital stay and 30-day case fatality rate.


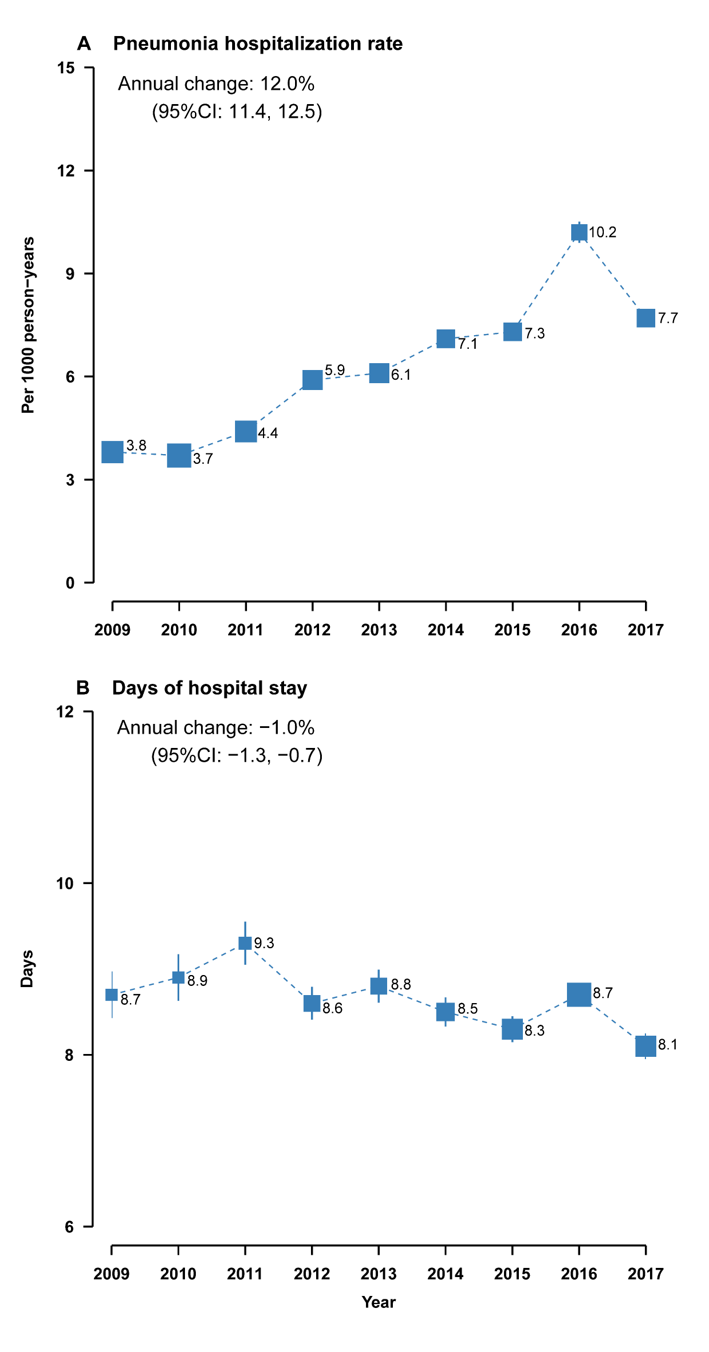


Supplementary Figure 4. Sensitivity analyses for secular trend by restricting analysis in the first-ever hospital admission for pneumonia

The data was adjusted for annually updated age, sex, and study area. Age was updated at the start of each year from 2009 to 2017 in the analysis of hospitalization rate or until the occurrence of the index hospitalization in the analysis of length of hospital stay and 30-day case fatality rate. The area of each square is inversely proportional to the variance, and 95% confidence intervals are shown. Numbers alongside the squares are hospitalization rates per 1000 person-years or mean length of hospital stay in days, as appropriate.


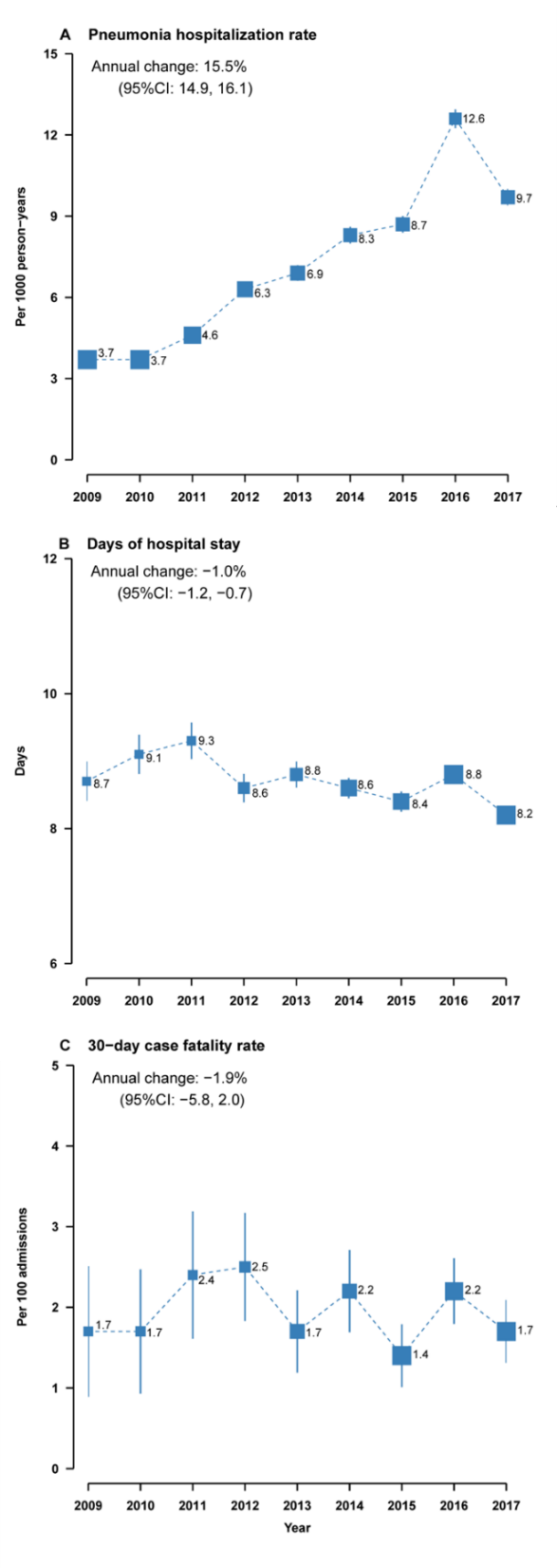


**Supplementary Figure 5. Sensitivity analyses for secular trend by restricting analysis in participants without any other hospital admission in the previous 30 days**

The data was adjusted for annually updated age, sex, and study area. Age was updated at the start of each year from 2009 to 2017 in the analysis of hospitalization rate or until the occurrence of the index hospitalization in the analysis of length of hospital stay and 30-day case fatality rate. The area of each square is inversely proportional to the variance, and 95% confidence intervals are shown. Numbers alongside the squares are hospitalization rates per 1000 person-years, mean length of hospital stay in days, or case fatality rates per 100 admissions, as appropriate.


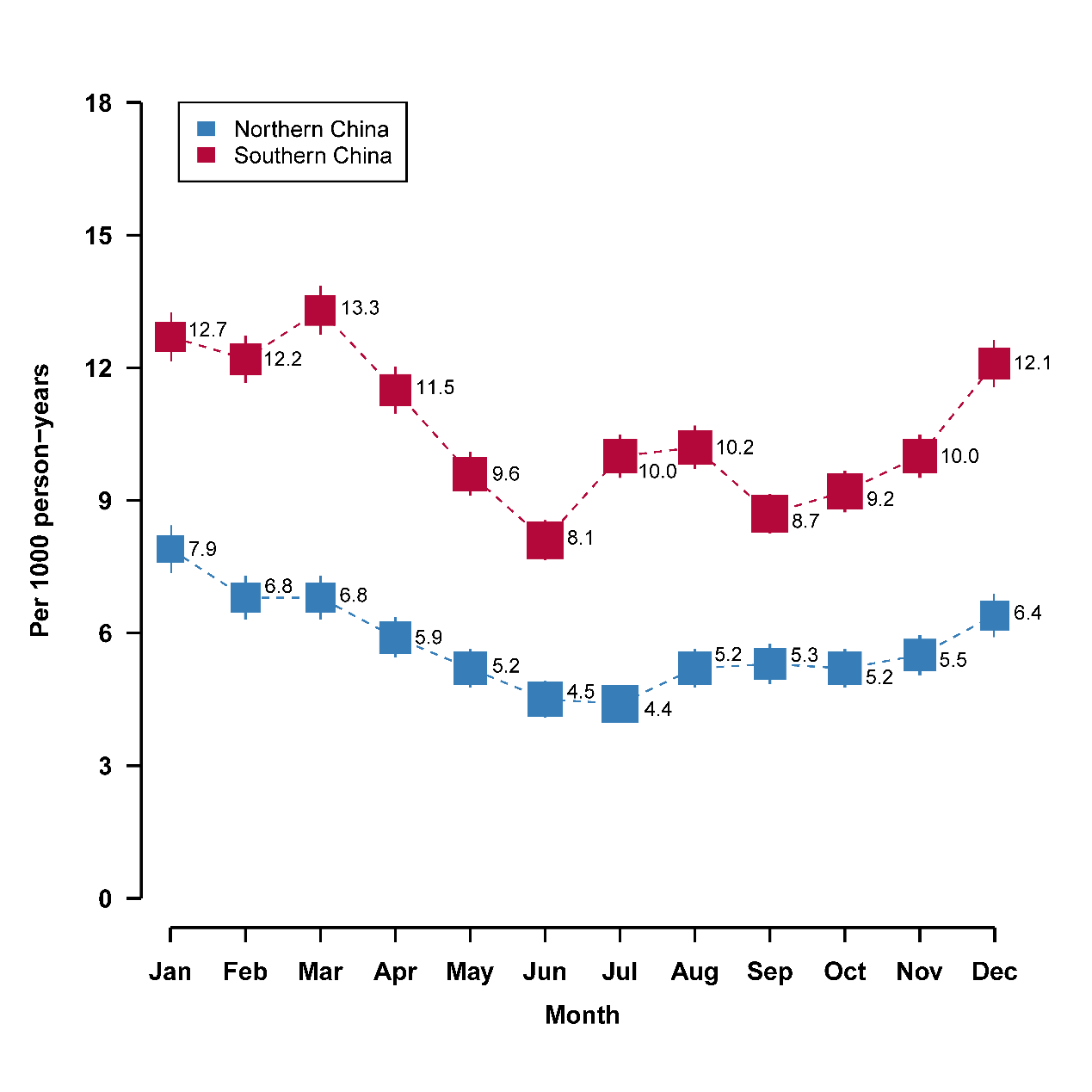
Supplementary Figure 6. Seasonal variation in pneumonia hospitalization from 2009 to 2017

We classified the study areas in Harbin, Qingdao, Gansu, and Henan as northern China and the other areas including Haikou, Suzhou, Liuzhou, Sichuan, Hunan, and Zhejiang as southern China according to the Qinling Mountain-Huaihe River Line. The data were adjusted for age at 2009, sex, and study area. The area of each square is inversely proportional to the variance, and 95% confidence intervals are shown. Numbers alongside the squares are hospitalization rates per 1000 person-years.


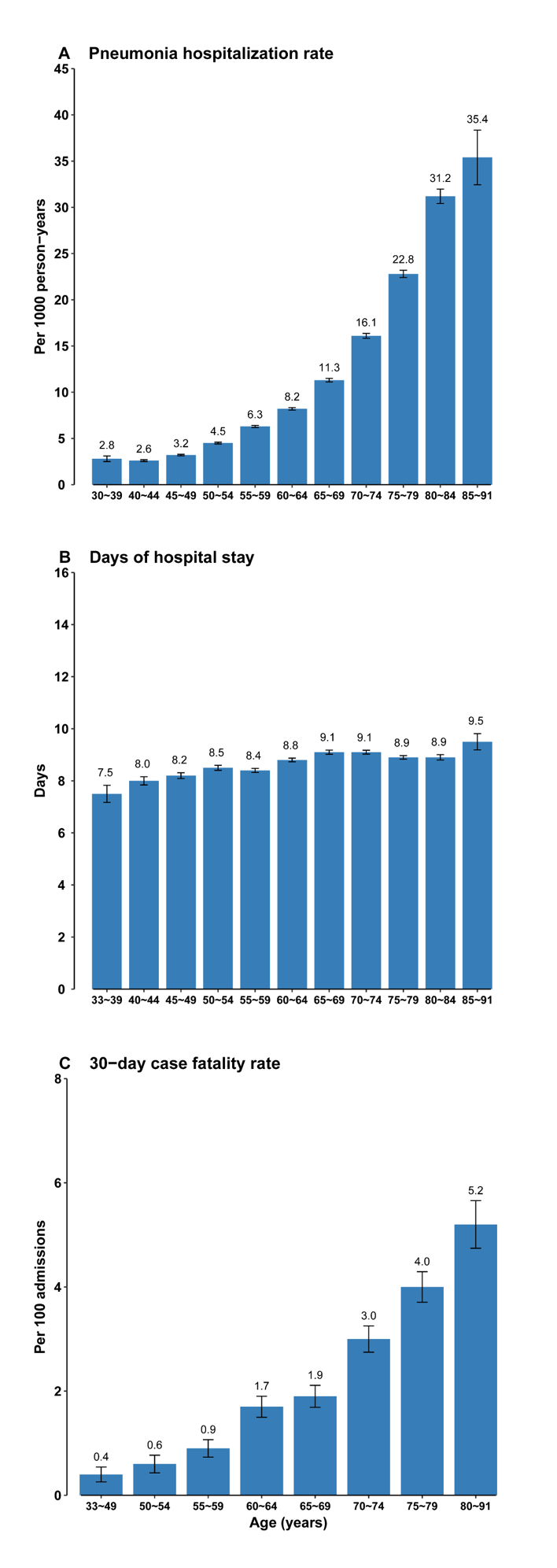


**Supplementary Figure 7. Variations in pneumonia hospitalization by age groups**

The data was adjusted for sex, study area, and the year of the index hospital admission. The error bar represents the standard error. Numbers above the histograms are rates per 1000 person-years, mean length of stay in days, or case fatality rates per 100 admissions, as appropriate.
