# Supplementary material for: The hospitalization burden of all-cause pneumonia in China: A population-based study, 2009–2017
Source: Lancet Reg Health West Pac. 2022 Apr 6;22:100443. doi: 10.1016/j.lanwpc.2022.100443 (PMC8991381; doi:10.1016/j.lanwpc.2022.100443)
Supplement: Supplementary file 2 [file mmc2.docx]

This translation in Chinese was submitted by the authors and we reproduce it as supplied. It has not been peer reviewed. Our editorial processes have only been applied to the original abstract in English, which should serve as reference for this manuscript.

**摘要**

**背景：**肺炎是一个重要的公共卫生问题，威胁人群健康的同时还造成沉重的经济负担。然而，基于人群研究的中国成年人肺炎住院负担的研究证据十分有限。

**方法:**中国慢性病前瞻性研究（China Kadoorie Biobank）于2004-2008年募集了来自5个城市和5个农村地区的50余万30-79岁的成年人。本研究纳入506,086名在2009年1月1日仍存活的研究对象。研究通过链接医保数据库获取研究对象2009年1月1日至2017年12月31日的肺炎住院数据。采用广义线性模型分析肺炎住院率、肺炎住院患者的住院时长及30天病死率的长期趋势及其地区、人群分布情况。

**结果:**在平均8.9年的随访期内，27,879名研究对象发生肺炎住院，合计发生肺炎住院36,567人次。研究期间内肺炎的粗住院率为8.4 (95% CI：8.3, 8.6)/1000人年，在调整年龄、性别和地区后，由2009年的4.2 (3.9, 4.4）增长至2017年的10.9 (10.6, 11.3)，平均年增长率为15.5%。研究期间肺炎住院患者平均住院时长为8.8（95% CI：8.7, 8.9）天，平均年缩短率为1.0%。肺炎住院患者的30天病死率没有明显改变，研究期间粗病死率为2.4（95% CI：2.2, 2.5)/100例肺炎住院。本研究还发现肺炎住院率存在明显的季节性；不同地区、年龄和基础疾病的患者，肺炎住院率和30天病死率都存在差异。

**解读:**中国成年人中肺炎住院负担重且呈现增长趋势，尤其是在≥60岁或存在基础疾病的研究对象中。
